# Unique 1D Co_3_O_4_ crystallized nanofibers with (220) oriented facets as high-performance lithium ion battery anode material

**DOI:** 10.1038/srep26460

**Published:** 2016-05-24

**Authors:** Yanli Tan, Qiuming Gao, Zeyu Li, Weiqian Tian, Weiwei Qian, Chunxiao Yang, Hang Zhang

**Affiliations:** 1 Key Laboratory of Bio-inspired Smart Interfacial Science and Technology of Ministry of Education, Beijing Key Laboratory of Bio-inspired Energy Materials and Devices, School of Chemistry and Environment, Beihang University, Beijing 100191, P. R. China

## Abstract

A novel one-step hydrothermal and calcination strategy was developed to synthesize the unique 1D oriented Co_3_O_4_ crystal nanofibers with (220) facets on the carbon matrix derived from the natural, abundant and low cost wool fibers acting as both carbon precursor and template reagent. The resultant W2@Co_3_O_4_ nanocomposite exhibited very high specific capacity and favorable high-rate capability when used as anode material of lithium ion battery. The high reversible Li^+^ ion storage capacity of 986 mAh g^−1^ was obtained at 100 mA g^−1^ after 150 cycles, higher than the theoretical capacity of Co_3_O_4_ (890 mAh g^−1^). Even at the higher current density of 1 A g^−1^, the electrode could still deliver a remarkable discharge capacity of 720 mAh g^−1^ over 150 cycles.

Rechargeable lithium ion batteries (LIBs) are widely considered to be one of the most promising power sources for the rapid development of hybrid electric vehicles and/or full electric vehicles as well as portable electronic devices, owing to their unique characteristics in terms of large energy density, long cyclic life and high operating voltage^1^,^2^,^3^,^4^,^5^,^6^. However, the energy and power densities of current generation LIBs are still limited by the electrode materials. Transition-metal oxides were proposed as the novel alternative anode materials, in which Co_3_O_4_ has attracted much more attention due to its promising properties, such as low-cost, environmental friendliness, high theoretical capacity (890 mAh g^−1^) compared to the commercialized carbon anode materials^7^,^8^,^9^,^10^,^11^,^12^. Nevertheless, the intrinsically slow ionic and electronic transport properties of Co_3_O_4_ may result in a large irreversible capacity loss and poor cyclic stability.

Various approaches have been adopted to improve the electrochemical performance of Co_3_O_4_, such as carbon coating, ion doping and nanostructure morphology control^13^,^14^,^15^,^16^,^17^,^18^. Besides, many researches have revealed that the crystal facet structures of electrode materials are important for the electrochemical energy storage properties^19^,^20^,^21^,^22^,^23^, however, the reactive high surface energy facets may lead to the aggregation and/or growth of the crystals. So, it is hard to obtain the Co_3_O_4_ nanocrystals with high surface energy in the equilibrium state or via the routine methods. Recently, several kinds of one-dimensional (1D) porous nanostructured materials were prepared and good properties were obtained as the LIB electrode active materials^24^,^25^,^26^,^27^,^28^. It can be deduced that the Co_3_O_4_ materials possessing of both well oriented high surface energy facets and suitable 1D morphology structures would exhibit the improved lithium storage properties as the anode materials of LIBs. Herein, unique 1D Co_3_O_4_ crystal nanofibers with (220) oriented facets on the carbon matrix derived from wool fibers (named as W2@Co_3_O_4_) were fabricated by one-step hydrothermal method following with calcination. When used as anode material of LIB, the W2@Co_3_O_4_ composite can deliver excellent reversible specific capacity, high rate performance and long cyclic capability.

## Results

**Figure 1:**
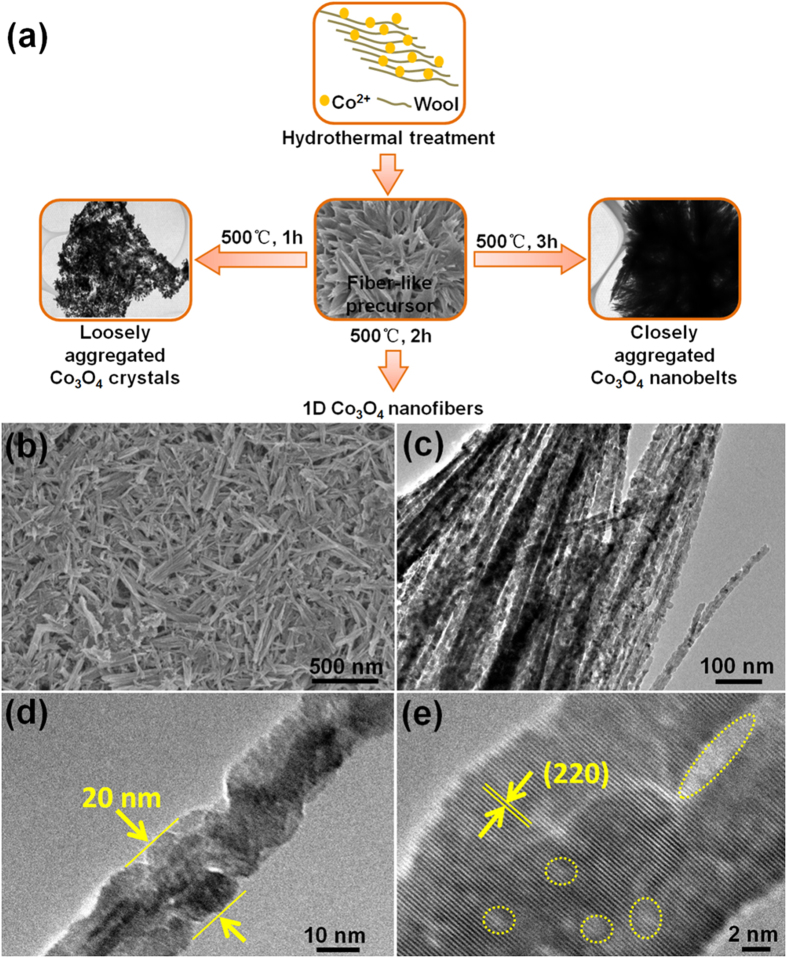
Synthesis, morphology and microstructure of the 1D W2@Co_3_O_4_ nanofibers. (**a**) Schematic illustration for the synthesis of W@Co_3_O_4_ composite, which inlustrates the growth process of the sample. (**b**) SEM of the W2@Co_3_O_4_ composite, indicating an identical 1D fiber-shaped morphology. (**c**,**d**) TEM images with different amplifications of the W2@Co_3_O_4_ composite, which consists of continuous and uniform 1D nanofibers. The nanofibers are as long as about 1 μm with almost constant diameter of about 20 nm. And (**e**) HRTEM image of the W2@Co_3_O_4_ composite, which shows well-crystallized nanocrystals with (220) oriented facets of Co_3_O_4_. The pores or vacancy defects are indicated by the circles in (**e**).

Schematic illustration for the synthesis of W@Co_3_O_4_ is shown in Fig. 1a. The natural 1D polymer wool fibers mainly made up of keratin were chosen as the carbon precursor and template. Scanning electron microscopy (SEM) images indicate that the diameter of the 1D polymer wool fibers is about 100 μm (Fig. S1a) and a certain degree scaling could be observed on the surface of the wool fibers (Fig. S1b). The fabrication of 1D W2@Co_3_O_4_ nanofibers was facilely controlled by adjusting the calcination time for the precursor obtained from the hydrothermal treatment. The synthesis strategy is as follows: In the first step, Co^2+^ ions were introduced into the solution containing wool fibers and deionized water with a thorough mixing process, which enables a full adsorption of Co^2+^ ions on the surface of wool substrate through charge attraction between the positively charged cobalt ions and the negatively charged functional groups of proteins in the wool. In the second step, a certain amount of urea was added into the above mixture, resulting in the formation of cobalt oxide precursor with a fiber-like morphology. The hydrothermal reaction brought about the formation of a fiber-like precursor, and the subsequent heating treatment under air at 500 °C for 2 h involved thermal decomposition of the precursor, resulting in the fabrication of 1D W2@Co_3_O_4_ nanofibers with hierarchically porous structure. SEM image (Fig. 1b) indicates that the resulting W2@Co_3_O_4_ sample has an identical 1D fiber-shaped morphology. TEM image (Fig. 1c) shows that the W2@Co_3_O_4_ sample consists of continuous and uniform 1D nanofibers, which are as long as about 1 μm with almost constant diameter of about 20 nm (Fig. 1d). The lattice fringes from the HRTEM image (Fig. 1e) of W2@Co_3_O_4_ show well-crystallized nanocrystals with lattice interplane spacing of 0.285 nm corresponding to the (220) facets of Co_3_O_4_. Moreover, several pores or vacancy defects may be found in the W2@Co_3_O_4_ nanofibers owing to thermally driven contraction force. Short calcination time (1 h) may result in the formation of W1@Co_3_O_4_, which is composed of the loosely aggregated Co_3_O_4_ crystals with the crystal sizes distributed in a wide range from about 20 to 200 nm (Fig. S2). The W3@Co_3_O_4_ sample obtained by the long calcination time (3 h) exhibits the closely aggregated bundles of Co_3_O_4_ nanobelts (Fig. S3). The C-content of W1@Co_3_O_4_, W2@Co_3_O_4_ and W3@Co_3_O_4_ composite was 72.84, 11.51 and 6.97 wt.%, respectively, based on the TGA measurements (Fig. S4).

**Figure 2:**
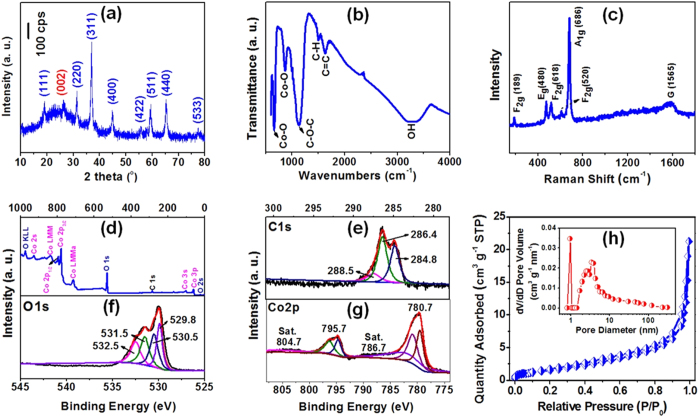
Structure and texture characterization of the 1D W2@Co_3_O_4_ nanofibers. (**a**) XRD patterns of the W2@Co_3_O_4_ composite. No impurity phase is found expect for carbon and Co_3_O_4_. (**b**) FT-IR spectrum, illuminating the formation of the W2@Co_3_O_4_ nanofibers. (**c**) Raman spectrum, demonstrating the formation of the carbon matrix derived from wool fibers and Co_3_O_4_. (**d**) XPS survey spectrum and high resolution XPS spectra of the W2@Co_3_O_4_ composite, which indicate the existence of carbon, oxygen and cobalt elements. (**e**) The high-resolution spectrum of the C 1s region, which could be split into three peaks: the sp^2^ bonded carbon at 284.8 eV (C-C/C = C), the epoxy at 286.4 eV (C-OH/C-OCo) and the carbonyls at 288.5 eV (HO-C=O). (**f**) The high-resolution spectrum of the O 1s region, comprising three peaks at 532.5 (C-OH), 531.5 (O-C) and 530.5 eV (O = C-OH). (**g**) The high-resolution XPS spectrum of the Co 2p, which shows two major peaks with the binding energy at 780.7 and 795.7 eV, corresponding to the Co 2p_3/2_ and Co 2p_1/2_ spin-orbit peak of Co_3_O_4_, respectively. And (**h**) N_2_ adsorption-desorption isotherms, indicating the high specific area and hierarchically porous structure. The inset is pore size distribution, showing a narrow pore size distribution with the pore size of about 1 nm and a broad pore size distribution mainly from 2–10 nm with the average pore size of 3.4 nm.

XRD patterns (Fig. 2a) demonstrate that the 1D W2@Co_3_O_4_ nanofibers have significant diffraction peaks with the 2θ value of 18.99°, 31.46°, 36.94°, 44.87°, 55.61°, 59.47°, 65.19° and 77.42°, which is corresponding to the (111), (220), (311), (400), (422), (511), (440) and (533) facet of the standard Co_3_O_4_ (JCPDS card no. 42-1467), respectively^29^. Besides, a broad and weak diffraction peak appearing at 2θ = 24.7° could be observed for W2@Co_3_O_4_, which can be indexed into the (002) facet of the carbon matrix with the hexagonal graphitic lattice derived from the high temperature calcination of the wool fibers in the precursor.

FT-IR spectra in the range of 4000–500 cm^−1^ are given to illuminate the formation of the W2@Co_3_O_4_ nanofibers (Fig. 2b). The distinct and sharp absorption peak at 661 cm^−1^ was observed, which may be assigned to the stretching vibration of the Co-O bond confirming the formation of the spinel Co_3_O_4_ in the composite. The sharp peak at 867 cm^−1^ is due to the asymmetric O-C-O bond. The existence of Co-O and O-C bonds indicates the close contact between Co_3_O_4_ and C in the composite, where the O element acts as the bridge between Co and C. The closely contacted Co_3_O_4_ and C could be favourable for the structure stability of the W2@Co_3_O_4_ composite. The peak at 3205 cm^−1^ is attributed to the O-H stretching vibrations. The peak at 1503 and 1133 cm^−1^ is corresponding to the carboxylic O-H deformation vibration and the C-O-C stretching vibration, respectively^30^,^31^,^32^. The peak at 1632 cm^−1^ can be assigned to the carbon skeletal vibrations (aromatic C = C) of the furanic and aromatic groups^29^.

Raman spectrum of the W2@Co_3_O_4_ nanofibers was measured to investigate the formation of the carbon matrix derived from wool fibers and Co_3_O_4_, showing five peaks at 189, 480, 520, 606 and 686 cm^−1^ (Fig. 2c). The peak at 189 cm^−1^ is attributed to the characteristic species of F_2g_^(3)^ symmetry in the tetrahedral sites (CoO_4_). The peak with medium intensity located at 480 and 520 cm^−1^ is corresponding to the species of E_g_ and F_2g_^(2)^ symmetry, respectively^33^. The weak peak located at 606 cm^−1^ is related to the species of F_2g_^(1)^ symmetry. The peak at 686 cm^−1^ is assigned to the species of A_1g_ in the O_h_^7^ spectroscopic symmetry, which is in consistent to the characteristic of the octahedral sites (CoO_6_)^34^,^35^. The observed five peaks are characteristics of the cubic Co_3_O_4_ phase, which is consistent with the XRD analyses. In addition, the broad peak displaying at 1565 cm^−1^ is assigned to the G-band of carbon, corresponding to the E_2g_ phonon of C sp^2^ atoms. The high intensity of the G-band compared to the D-band indicates a good graphitization of the carbon matrix derived from wool fibers, where the more sp^2^-hybridized carbon atoms formed in stacked and highly ordered hexagonal rings may give rise to the enhanced conductivity of the carbon matrix.

XPS measurements were carried out to determine the chemical composition and elemental valence state of the W2@Co_3_O_4_ nanofibers. XPS survey scan spectrum of W2@Co_3_O_4_ (Fig. 2d) exhibits the characteristic peak at 284.6, 529.6 and 779.9 eV, corresponding to the C 1s, O 1s and Co 2p component, respectively. The C 1s (Fig. 2e) could be split into three peaks: the sp^2^ bonded carbon at 284.8 eV (C-C/C = C), the epoxy at 286.4 eV (C-OH/C-OCo) and the carbonyls at 288.5 eV (HO-C = O), indicating the nonoxygenated carbon atom, hydroxyl groups and oxygen containing functional groups^29^. Figure 2f shows the XPS spectrum of the O 1s, comprising three peaks at 532.5 (C-OH), 531.5 (O-C) and 530.5 eV (O = C-OH). The peak at the lower energy of 529.8 eV is associated with the lattice oxygen in the spinel Co_3_O_4_^36^. The Co 2p XPS spectrum (Fig. 2g) shows two major peaks with the binding energy at 780.7 and 795.7 eV, corresponding to the Co 2p_3/2_ and Co 2p_1/2_ spin-orbit peak of Co_3_O_4_, respectively^29^. The binding energy difference between the two peaks is 15 eV and their intensity ratio is almost 2:1, which are typical characteristics of the standard Co_3_O_4_ spectrum. The linkage between Co-O and C-O brings about a large electron charge overlap in the interface between the Co_3_O_4_ and carbon matrix derived from wool fibers, which may promote the electronic transports during the charge-discharge cycles.

N_2_ adsorption-desorption measurements were performed to give the porous characteristics of the W2@Co_3_O_4_ nanofibers (Fig. 2h). The Brumauer-Emmett-Teller (BET) specific area and total pore volume was calculated to be 78.25 m^2 ^g^−1^ and 0.393 cm^3 ^g^−1^, respectively (Fig. 2h). A narrow pore size distribution with the pore size of about 1 nm and a broad pore size distribution mainly from 2–10 nm with the average pore size of 3.4 nm were confirmed for the W2@Co_3_O_4_ nanofibers by the pore size distribution curve (inset in Fig. 2h) analyses based on the density functional theory model. It can be clearly seen that the hierarchically porous W2@Co_3_O_4_ delivers the highest surface area among all the investigated W@Co_3_O_4_ samples (Fig. S5 and Table S1).

**Figure 3:**
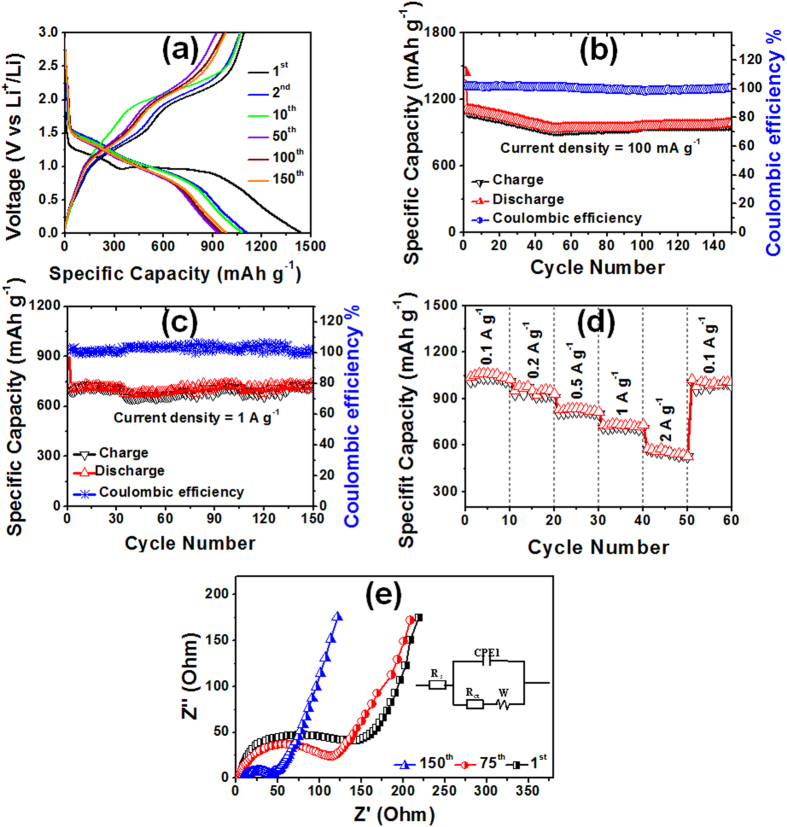
Electrochemical properties of the W2@Co_3_O_4_ composite anode for LIB. (**a**) Galvanostatic charge/discharge profiles for the 1^st^, 2^nd^, 10^th^, 50^th^, 100^th^ and 150^th^ cycle at 100 mA g^−1^. (**b**) Plots of charge-discharge capacities versus cycle number and the Coulomb efficiency at a current density of 100 mA g^−1^ between 0.01 and 3.0 V. (**c**) Capacity vs. cycle number and the corresponding Columbic efficiency at a current density of 1 A g^−1^. (**d**) Rate performance at various current densities from 0.1 to 2 A g^−1^ in the voltage range of 0.01–3.0 V. And e, the Nyquist plots for the W2@Co_3_O_4_ nanofibers after 1, 75 and 150 cycles with the inset of the simulation model of the equivalent circuit.

The charge-discharge properties of the W2@Co_3_O_4_ anode materials were determined at a current density of 100 mA g^−1^ (Fig. 3a,b). The first discharge and charge capacity was 1442 and 1092 mAh g^−1^, respectively, corresponding to the Coulombic efficiency of 75.7%. The long discharge voltage plateau at 0.95–1.0 V could be associated with the reduction process from Co_3_O_4_ to metallic cobalt^14^. The discharge specific capacity was 1112 mAh g^−1^ at the 2^nd^ cycle and gradually decreased to 937 mAh g^−1^ at the 50^th^ cycle (Fig. 3b), where the Coulombic efficiency of W2@Co_3_O_4_ quickly increased to 95.6% for the 2^nd^ cycle and stabilized in the range of 96–99% in the subsequent cycles. The decreased capacity could be dominated by the decreasing conductivity caused by the phase transformation and pulverization of the electrode materials upon cycling^37^. After that, the specific capacity continuously increases with the high specific capacity of 986 mAh g^−1^ at the 150^th^ cycle with the stabilized Coulombic efficiency of 96–99%. As for W2@Co_3_O_4_, the unique 1D nanostructure with the high surface energy (220) oriented facets may result in easy transports of Li^+^ ions, and the carbon matrix from wool fibers with the good graphitization could mitigate the volume expansion. Therefore, these factors jointly weakened phase transformation and particle pulverization, bringing about the unusual continuously increased capacity from the 50^th^ to the 150^th^ cycle. The CV curves of the W2@Co_3_O_4_ electrode (Fig. S6) are in good agreement with the charge-discharge voltage profiles. For comparison, the specific capacities of W1@Co_3_O_4_ and W3@Co_3_O_4_ electrodes were also determined and low specific capacities were obtained after the same cycling numbers (Figs S7 and S8). Apparently, the W2@Co_3_O_4_ revealed the best electrochemical performance with high specific capacity and excellent cyclic stability in all the three composite electrodes.

Because the rate capability is an important parameter for the LIB anode material, the cyclic stability of the W2@Co_3_O_4_ electrode was evaluated at a high current rate of 1 A g^−1^ (Fig. 3c). The specific capacity was still kept 720 mA h g^−1^ at 1 A g^−1^ for 150 cycles, indicating a superior rate capability of the W2@Co_3_O_4_ electrode. The Coulombic efficiency of the W2@Co_3_O_4_ electrode at 1 A g^−1^ corresponds to 100% (Fig. 3c), further indicating its high electrochemical stability. The rate performance of W2@Co_3_O_4_ at various current rates was investigated (Fig. 3d). The cells cycled at 100 mA g^−1^ for 50 cycles were used for rate capability test in order to avoid the induced effect due to the activation of the electrode. A reversible capacity of 1042 mAh g^−1^ was achieved at 100 mA g^−1^. When the charge-discharge rate increased to the 500 mA g^−1^, the W2@Co_3_O_4_ electrode could still exhibit a reversible capacity of 828 mAh g^−1^. Even at high rate of 1 and 2 A g^−1^, the electrode reserved a specific capacity of 733 and 580 mAh g^−1^, respectively. Moreover, when the current density returned to the 100 mA g^−1^, the discharge capacity recovered to the same levels initially shown at that rate. Notably, the result is much better than most of the reported Co_3_O_4_-based anode materials, such as peapod-like Co_3_O_4_/carbon nanocomposites^38^, graphene-anchored Co_3_O_4_ nanoparticle^39^, Co_3_O_4_/CNT heterostructures^40^, graphene-encapsulated mesoporous Co_3_O_4_ microspheres^41^, graphene-coated Co_3_O_4_ fibers^42^, and Co_3_O_4_/carbon composite nanowires^43^. It should be mentioned that the result is still superior to that of our reported 1D H2@Co_3_O_4_ nanofiber electrode^26^. For clarity, the electrochemical performances of W2@Co_3_O_4_ and the reported Co_3_O_4_-based materials have been summarized in Table S2. As for W2@Co_3_O_4_, the carbon matrix derived from natural, abundant and low cost wool, which is a readily available waste and could provide the potential to large scale production of the electrode.

**Figure 4:**
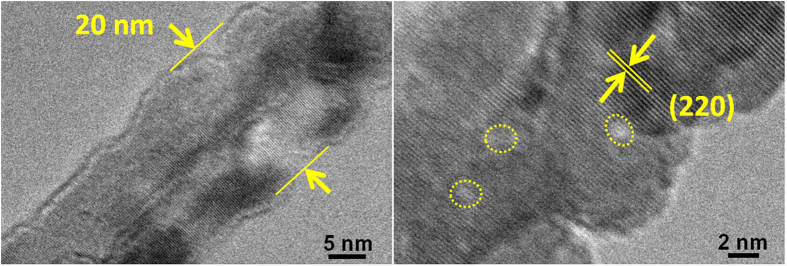
The high structural stability of the W2@Co_3_O_4_ composite after 150 cycles at a current density of 0.1 A g^−1^. (**a**) TEM and (**b**) HRTEM images of W2@Co_3_O_4_ after 150 cycles at a current density of 0.1 A g^−1^. No obvious changes in the morphology, size and microstructure could be observed for the W2@Co_3_O_4_ composite, indicating the structural integrity of the composite upon electrochemical cycling.

The electrochemical impedance spectroscopies (EIS) were determined to understand the enhanced rate capability. The Nyquist plots for W2@Co_3_O_4_ at different cycles from 0.1 MHz to 0.01 Hz are shown in Fig. 3e. All the curves describe a semicircle at high-medium frequency and an inclined line at low frequency, which respectively correspond to the charge transfer and diffusion. The equivalent circuit is indicated as the insert of Fig. 3e, where, R_s_ is the ionic resistance of electrode (intrinsic resistance of substrate, and contact resistance at the active material/current collector interface), R_ct_ is the charge transfer resistance (the semicircle diameter), Z_W_ is the Warburg impedance (Li^+^ ions diffusion into the active materials, slope of the curve at low frequency), and CPE is the constant phase-angle element which involves the double layer capacitance^31^. It can be clearly seen that the R_ct_ became lower from 146.1, 113.7 to 40.7 Ω with the increased cycling after 1, 75 and 150 cycles, demonstrating the good electrolyte infiltration and charge-transport capability. In the low frequency region, the W2@Co_3_O_4_ electrode showed less Z_w_ with a line slope close to 45^o^, which is a result of Li^+^ ions diffusion dependence at electrolyte/electrode interface^44^. For comparison, the Nyquist plots of the W1@Co_3_O_4_ and W3@Co_3_O_4_ electrodes are shown in Fig. S9. Apparently, the R_ct_ of W2@Co_3_O_4_ nancomposites was smaller than that of the other two electrodes, resulting from the 1D nanofiber porous structure. The above mentioned EIS results suggest that the W2@Co_3_O_4_ nanofibers have the lowest activation energy for the Li^+^ ions diffusion and undergo a fast Faradaic reaction. Besides, the (HR)TEM images (Fig. 4) of the W2@Co_3_O_4_ electrode after 150 cycles were also determined. No obvious changes can be observed on the size, shape and microstructure of the W2@Co_3_O_4_ composite, indicating the structural integrity of the composite after the electrochemical cycling.

## Discussions

The unique 1D porous W2@Co_3_O_4_ nanocomposite possessing of the oriented Co_3_O_4_ crystal nanofibers with the (220) facets on the carbon matrix has been successfully synthesized via one-step hydrothermal method following with calcination. The formation of well-defined 1D nanosized morphology can be facilely controlled by adjusting the calcination time of the precursor obtained from the hydrothermal treatment. The natural, abundant and low-cost wool fibers act as both the precursor of the carbon matrix and template reagent of the W2@Co_3_O_4_ nanofibers. The W2@Co_3_O_4_ product can deliver excellent reversible capacity, high rate performance and long cyclic stability used as the anode material of LIBs due to the following reasons: (I) the oriented (220) facets with much higher energies could reduce the oxidation-reduction gaps, thus greatly accelerating the reaction rates; (II) the 1D nanostructure with hierarchical pores may effectively reduce the Li^+^ ions diffusion lengths and provide space for volume shrinking and expansion during insertion/extraction processes resulting in improved Li^+^ ion diffusion rate and high cyclic stability; (III) the carbon matrix from wool fibers with the good graphitization could enhance the electron transports and mitigate the volume expansion; and (IV) the close contact between Co_3_O_4_ and C could be favorable for the structure stability of the W2@Co_3_O_4_ composite. These factors simultaneously provide the advantages of the 1D W2@Co_3_O_4_ nanofibers as the high-performance Li^+^ ion storage materials and they are very important for the rational design of advanced electrode material of LIBs.

## Methods

### Materials preparation

The wool fibers obtained from Zhejiang province of China were thoroughly washed with isopropanol and dried at 80 °C. The cleaned fibers were cut into fine debris (~5 mm in length). All the chemicals used in the experiments are analytical grade and were used without further purification. The precursors are synthesized under hydrothermal condition. In a typical synthesis, 2.0 mmol of Co(CH_3_COO)_2_·4H_2_O was dissolved in 40 mL of a mixture containing 3.0 mL of ethylene glycol and 37 mL of deionized water. After stirring for 15 min, a certain amount of urea was added into the above solution. The mixture was stirred for another 30 min. Then 0.5 g of cleaned wool fibers were added into the above solution and immersed for 1 h. The obtained mixture was transferred into a 50 mL Teflon-lined stainless steel autoclave. The autoclave was sealed and maintained at 200 °C for 24 h in an electron oven. After that, the autoclave was cooled naturally to room temperature. The product was collected and washed with deionized water and ethanol for several times by centrifugation, followed by vacuum-drying at 60 °C. After calcinating the collected precursor at 500 °C in air for different time (1, 2 and 3 h), three kinds of W@Co_3_O_4_ composites were obtained, which is accordingly named as W1@Co_3_O_4_, W2@Co_3_O_4_ and W3@Co_3_O_4_, respectively.

### Characterization

Scanning electron microscopy (SEM) measurements were carried out on JSM-7500F (5 kV) instrument. Transmission electron microscopy (TEM) and high-resolution TEM (HRTEM) were examined on JEOL JEM-2100F at an acceleration voltage of 200 kV. Powder X-ray diffraction (XRD) patterns were determined on the X-ray diffractometor (X-ray 6000) with the 2θ angle region from 10° to 80° at a scan rate of 3° min^−1^. Fourier transform infrared (FT-IR) spectra were obtained by the spectrophotometer (Nicolet iN10 MX, USA). Raman spectra were measured on a microscopic confocal Raman spectrometer (Lab RAM HR800) under a back scattering geometry (λ = 514 nm). X-ray photoelectron spectroscopy (XPS) analyses were performed using an Al K_α_ (150 W) monochromatic X-ray source (ESCALAB 250, Thermo Fisher Scientific, USA). N_2_ adsorption-desorption isotherms were examined at 77 K using a Micromeritics ASAP 2020. Thermogravimetric analyses (TGA) were determined at SDTQ600 (TA Instruments, USA) under an air atmosphere at a heating rate of 10 °C min^−1^ from room temperature to 700 °C. Cyclic voltammetry (CV) was performed by using CHI1040C electrochemical work station between 0.01 and 3.0 V at a scan rate of 0.2 mV s^−1^. The galvanostatic charging/discharging test was conducted by using coin cells (CR2032) at room temperature on a multi-channel battery testing system (LAND CT2001A) with a cutoff voltage of 0.01–3.0 V vs Li^+^/Li. Working electrodes were prepared by mixing 80 wt.% the resulting W@Co_3_O_4_ material, 10 wt.% acetylene black (Super-P) and 10 wt.% polyvinylidenefluoride (PVDF) binder dissolved in N-methyl-2-pyrrolidinone (NMP). 1.0 M LiPF_6_ in mixed ethylene carbonate (EC) and diethyl carbonate (DEC) (EC: DEC = 1:1 by volume) was used as the electrolyte in the system.

## Additional Information

**How to cite this article**: Tan, Y. *et al*. Unique 1D Co_3_O_4_ crystallized nanofibers with (220) oriented facets as high-performance lithium ion battery anode material. *Sci. Rep.* **6**, 26460; doi: 10.1038/srep26460 (2016).

## Supplementary Material

Supplementary Information
