# Supplementary material for: Unique 1D Co3O4 crystallized nanofibers with (220) oriented facets as high-performance lithium ion battery anode material
Source: Sci Rep. 2016 May 24;6:26460. doi: 10.1038/srep26460 (PMC4877706; doi:10.1038/srep26460)
Supplement: Supplementary Information [file srep26460-s1.doc]

*Supplementary information*

**Unique 1D Co_3_O_4_ crystallized nanofibers with (220) oriented facets as high-performance lithium ion battery anode material**

Yanli Tan^1^, Qiuming Gao[[1]](#footnote-2),* Zeyu Li^1^, Weiqian Tian^1^, Weiwei Qian^1^, Chunxiao Yang^1^, and Hang Zhang^1^


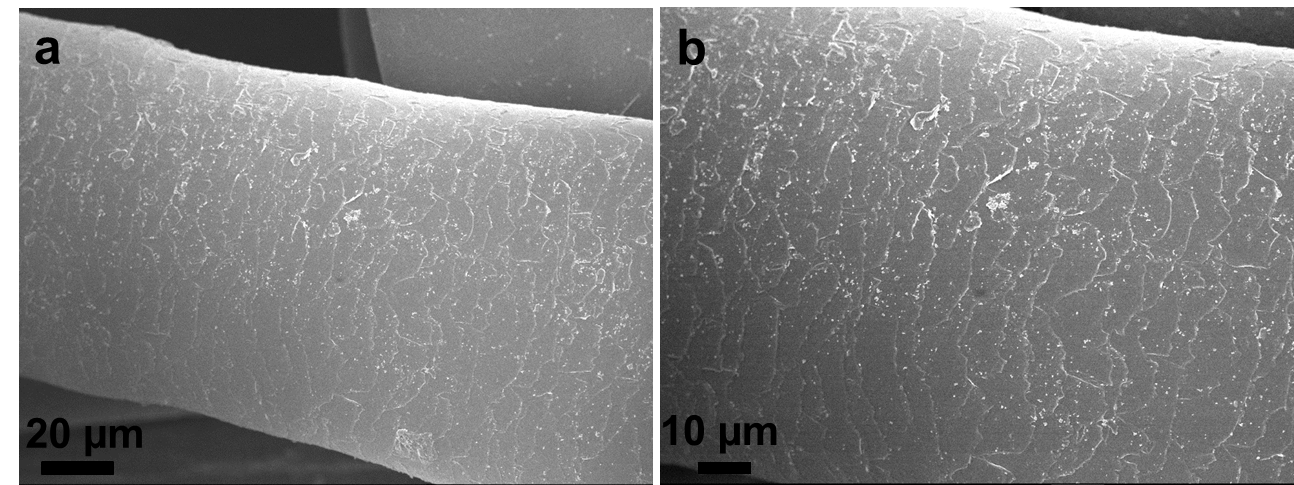


**Figure S1.** (a and b) SEM images of the wool ﬁber.

**
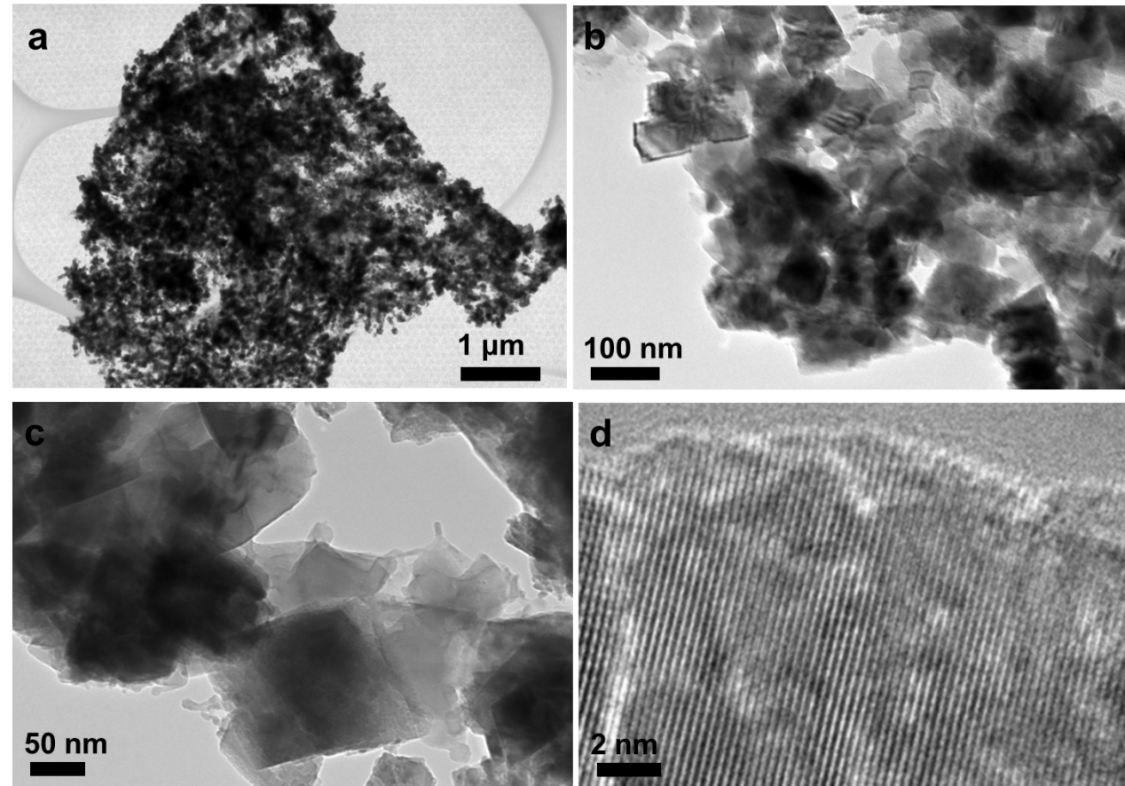
**

**Figure S2.** (a-c) TEM and HRTEM (d) of W1@Co_3_O_4_ prepared by hydrothermal treatment of the mixture of wool-Co(CH_3_COO)_2_·4H_2_O-urea-ethylene glycol-H_2_O following with high-temperature calcination at 500℃ in air for 1 h.


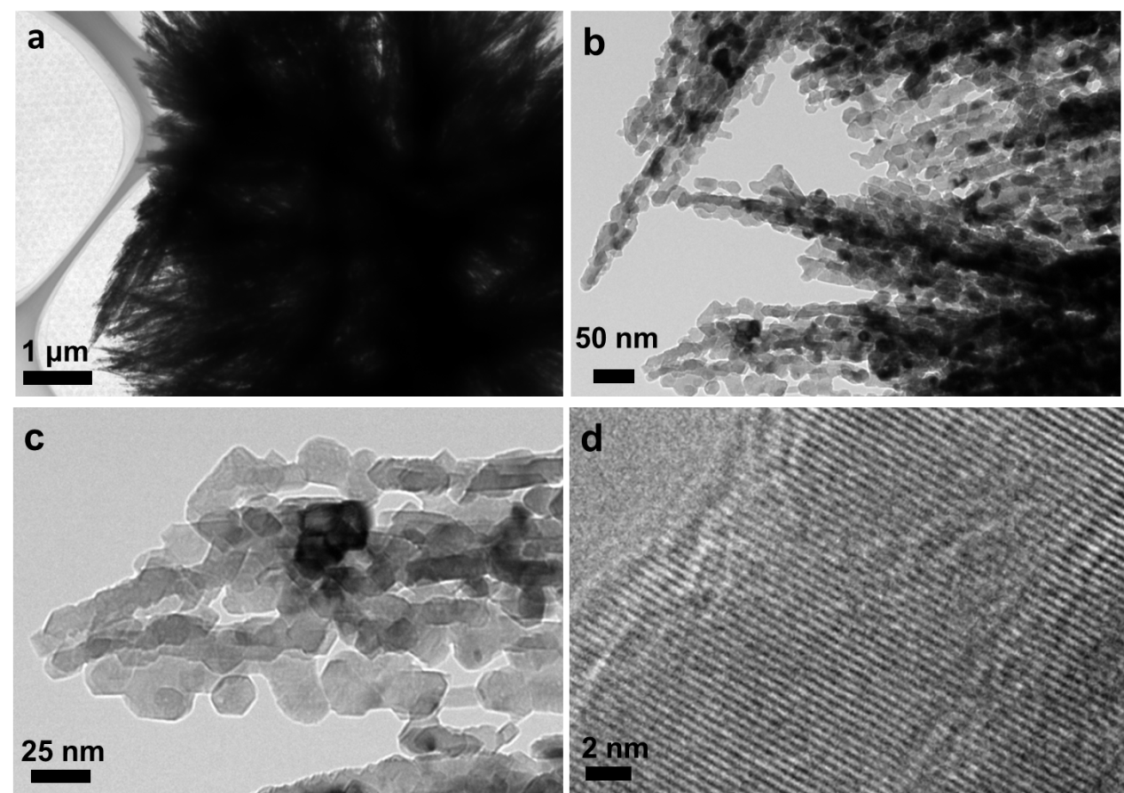


**Figure S3.** (a-c) TEM and HRTEM (d) of W3@Co_3_O_4_ prepared by hydrothermal treatment of the mixture of wool-Co(CH_3_COO)_2_·4H_2_O-urea-ethylene glycol-H_2_O following with high-temperature calcination at 500℃ in air for 3 h.

**Figure S4.** TGA curves of W@Co_3_O_4_ composites under an air atmosphere at a heating rate of 10℃ min^-1^ from room temperature to 700℃.

**Figure S5.** N_2_ adsorption-desorption isotherms and the inset pore size distribution curves of the (a) W1@Co_3_O_4_ and (b) W3@Co_3_O_4_ electrodes.

**Figure S6.** The representative CV curves of W2@Co_3_O_4_ nanofibers between 0.01 and 3.0 V at a scan rate of 0.2 mV s^-1^.

**Figure S7.** Charge-discharge profiles of the (a) W1@Co_3_O_4_ and (b) W3@Co_3_O_4_ electrodes for different cycles between 0.01 and 3.0 V at a current density of 100 mA g^-1^.

**Figure S8.** Charge-discharge capacities versus cycle number of W1@Co_3_O_4_, W2@Co_3_O_4_ and W3@Co_3_O_4_ electrodes at a current density of 100 mA g^-1^ between 0.01 and 3.0 V.

**Figure S9.** The Nyquist plots of W1@Co_3_O_4_, W2@Co_3_O_4_ and W3@Co_3_O_4_ electrodes from 0.1 MHz to 0.01 Hz.

**Table S1.** Pore textural parameters for the W@Co_3_O_4_ composites.

| Sample | S_BET_  [m^2^ g^-1^] ^a)^ | V_t_  [cm^3^ g^-1^] ^b)^ | D_BJH_  [nm] ^c)^ |
| --- | --- | --- | --- |
| W1@Co_3_O_4_ | 73.35 | 0.444 | 2.0 |
| W2@Co_3_O_4_ | 78.25 | 0.393 | 3.4 |
| W3@Co_3_O_4_ | 52.09 | 0.344 | 3.4 |

^a)^ S_BET_ represented the BET surface area; ^b)^ V_t_ was on behalf of the total pore volume; and ^c)^ D_BJH_ was representative of the BJH desorption average pore diameter.

**Table S2. The electrochemical performance of W2@Co_3_O_4_ nanofibers compared with the reported results on the Co_3_O_4_-based materials.**

| Material | Current density  [mA g^-1^] | The first discharge/charge capacity  [mAh g^-1^] | Initial Coulombic efﬁciency [%] | Cycle number | Capacity  [mAh g^-1^] | Ref. |
| --- | --- | --- | --- | --- | --- | --- |
| H2@Co_3_O_4_ | 100 | 1368/1031 | 75.4 | 100 | 916 | 26 |
| Co_3_O_4_@carbon | 890 | 1800/1050 | 58.3 | 50 | 800 | 38 |
| Co_3_O_4_/graphene | 58 | 1097/753 | 68.6 | 30 | 800 | 39 |
| Co_3_O_4_/CNT | 250 | 1248/878 | 70.3 | 30 | 758 | 40 |
| G-Co_3_O_4_ | 100 | 1533/1266 | 82.6 | 35 | 820 | 41 |
| GNS/Co_3_O_4_ paper | 100 | 1405/1006 | 71.6 | 40 | 850 | 42 |
| Co_3_O_4_/carbon | 100 | 1146/730 | 63.7 | 20 | 534 | 43 |
| W2@Co_3_O_4_ | 100/1000 | 1442/1092  938/700 | 75.7/74.6 | 150 | 986/720 | Our work |

1. Key Laboratory of Bio-inspired Smart Interfacial Science and Technology of Ministry of Education, Beijing Key Laboratory of Bio-inspired Energy Materials and Devices, School of Chemistry and Environment, Beihang University, Beijing 100191, P. R. China. Correspondence and requests for materials should be addressed to Q.-M.G. (E-mail: [qmgao@buaa.edu.cn](mailto:qmgao@buaa.edu.cn)). [↑](#footnote-ref-2)
